# Case Report: Congenital pulmonary airway malformation associated with a germline DICER1 splicing variant

**DOI:** 10.3389/fped.2026.1876103

**Published:** 2026-07-17

**Authors:** Ya Dao, Shan Pei, Xichen Zhang, Yunshan Gao, Rutao Dai, Xian Zhu, Yongyu Ma, Jun Zhou, Jun Wu, Qinghua Xu

**Affiliations:** 1 Department of Cardiothoracic Surgery, National Regional Children's Medical Center, Kunming Children’s Hospital, Yunnan Provincial Children’s Hospital, Children’s Hospital Affiliated to Kunming Medical University, Kunming, Yunnan, China; 2 Yunnan Institute of Pediatrics, National Regional Children's Medical Center, Kunming Children’s Hospital, Yunnan Provincial Children’s Hospital, Kunming, Yunnan, China; 3 Department of Pathology, National Regional Children's Medical Center, Kunming Children’s Hospital, Yunnan Provincial Children’s Hospital, Kunming, Yunnan, China

**Keywords:** congenital pulmonary airway malformation, DICER1 syndrome, germline variant, pleuropulmonary blastoma, splicing, tumor predisposition syndrome

## Abstract

**Background:**

Congenital pulmonary airway malformation type IV (CPAM IV) and pleuropulmonary blastoma (PPB) exhibit significant radiographic and histopathological overlap, making their differentiation challenging. While *DICER1* mutations are known to predispose individuals to the PPB spectrum, the molecular association between CPAM IV and early-stage PPB remains controversial.

**Case presentation:**

We report a girl pathologically diagnosed with CPAM IV. Whole-exome sequencing (WES) of the lesion tissue identified a heterozygous splicing variant in the *DICER1* gene (c.4206 + 1G > T). Sanger sequencing subsequently confirmed this variant to be germline, inherited from her asymptomatic father. Bioinformatic analysis predicted that this variant disrupts the highly conserved donor splice site of intron 22. Functional validation by RT-PCR demonstrated that the c.4206 + 1G > T variant results in exon fragment deletion. According to the ACMG/AMP guidelines, this variant was classified as likely pathogenic. Additionally, a somatic *DICER1* hotspot mutation (c.5438A > G p.E1813G) was detected in the tissue, consistent with the two-hit tumorigenesis model. At the 22-month postoperative follow-up, although chest CT revealed a small cystic lucency with surrounding calcification, the patient remained clinically stable, without evidence of malignant progression or extrapulmonary involvement.

**Conclusion:**

This article reports a case of CPAM IV carrying a pathogenic germline variant and a somatic hotspot mutation in the *DICER1* gene. Our findings support the view that DICER1-associated CPAM IV may represent an early stage within the PPB disease spectrum. Given the incomplete penetrance of DICER1 syndrome and an approximately 50% risk of transmission to offspring, we propose that *DICER1* genetic testing could be considered for selected pediatric patients diagnosed with CPAM IV, regardless of family history. This approach may aid in early and accurate differentiation, inform surgical management, and guide the development of appropriate long-term surveillance strategies.

## 1 Introduction

Congenital pulmonary airway malformation (CPAM), formerly known as congenital cystic adenomatoid malformation, represents a group of rare cystic lesions of the lower respiratory tract caused by bronchopulmonary developmental abnormalities (1). Stocker classified CPAM into types 0 to IV based on the level of airway developmental arrest or delay (2). CPAM is generally regarded as a benign developmental abnormality. However, with recent advances in molecular genetics, particularly regarding the biological nature of type IV lesions and their relationship with pleuropulmonary blastoma (PPB), new insights have emerged, along with ongoing controversies.

PPB is a rare and highly aggressive embryonal mesenchymal tumor of the lung (3). Its development is driven by pathogenic variants in the *DICER1* gene, which encodes an RNase III endoribonuclease essential for microRNA biogenesis (4). Pathogenic alterations in *DICER1* give rise to an autosomal dominant tumor predisposition syndrome known as DICER1 syndrome. This syndrome is characterized by incomplete penetrance and marked phenotypic heterogeneity. Its clinical manifestations extend beyond PPB to include cystic nephroma, ovarian Sertoli–Leydig cell tumors, multinodular goiter, and others (5).

Multiple studies have demonstrated that CPAM IV and PPB I (or its regressive subtype, Ir) exhibit substantial overlap in imaging and histopathological features, making reliable differentiation exceptionally difficult in clinical practice (6–8). Based on this marked morphological overlap, some investigators have proposed that the two lesions belong to the same clinicopathological spectrum (9). With accumulated clinicopathological and molecular evidence, the lesions formerly classified as CPAM IV should be regarded as PPB I (10). However, molecular and immunophenotypic studies suggest that these lesions may exhibit biological differences. CPAM has been reported to demonstrate upregulated expression of FGF10 signaling pathway-associated molecules, whereas PPB I typically lacks such expression. In addition, downregulation of YY1 expression and interstitial nuclear positivity of p53 are more commonly observed in PPB (11, 12). Current literature, notably within the PPB/DICER1 domain, indicates that in the presence of pathogenic germline *DICER1* mutations, CPAM IV-like imaging or pathological findings are unlikely to represent a stable independent benign endpoint. Instead, such lesions may represent the earliest and most indolent pulmonary manifestation of DICER1 tumor predisposition syndrome, corresponding to type I or regressed (type Ir) PPB (13). Therefore, elucidating the genotype-phenotype relationship is crucial for the early identification of potential malignancies, guiding genetic counseling, and developing appropriate clinical surveillance strategies.

Here, we report a pediatric case with imaging and pathological findings consistent with CPAM IV, in whom genetic testing identified both a germline mutation and a somatic hotspot mutation in the *DICER1* gene. By integrating detailed clinicopathological data and family history, we investigated the association between this variant and the cystic pulmonary lesions, with the aim of informing clinical differential diagnosis and long-term management.

## 2 Materials and methods

### 2.1 Subjects

The patient was a 6-year-10-month-old girl who was admitted for treatment due to an incidental finding of a left pulmonary cyst on imaging for more than 3 years. Her medical history was notable for tonsillectomy performed 2 years earlier for adenoid hypertrophy. Family history revealed no apparent abnormalities in her parents or sister, and both clinical evaluation and medical history indicated that they were healthy. Chest CT of the father showed no cystic lesions in the lungs, but incidentally revealed right-sided convexity of the T4-T5 thoracic vertebrae and elevation of the right hemidiaphragm. The study was approved by the Ethics Committee of Kunming Children's Hospital, and written informed consent was obtained from the patient's family.

### 2.2 Whole exome sequencing (WES) and data analysis

Genomic DNA was extracted from tissue samples using the nucleic acid extraction Kit (Magnetic Bead Method) (IVD0010, MyGenostics, China) according to the manufacturer's standard protocol. WES was performed by Beijing MyGenostics Co., Ltd. using the Illumina NovaSeq 6000 platform with paired-end 150 bp reads (PE150). Raw FASTQ data were subjected to quality control using Cutadapt software to obtain high-quality clean reads. The filtered reads were then aligned to the human reference genome (GRCh37/hg19). Variant calling and genotyping were conducted following the GATK best-practice pipeline, and all identified variants were functionally annotated using the ANNOVAR online tool. Candidate variants were filtered according to the following criteria: variants with a minor allele frequency (MAF) >0.1% in the gnomAD database were excluded; only variants located in exonic regions or canonical splice sites were retained; and particular attention was given to variants predicted to affect protein function, including missense, frameshift, and nonsense mutations. Clinical significance was assessed using the ClinVar and HGMD databases. All variants were classified according to the American College of Medical Genetics and Genomics and the Association for Molecular Pathology (ACMG/AMP) guidelines. Finally, potential pathogenic variants were validated in the proband and available family members through Sanger sequencing.

To evaluate the potential impact of the candidate variant on protein function and gene splicing, a comprehensive bioinformatic pipeline was implemented. The functional consequences of the identified variant were initially assessed using multiple in silico pathogenicity prediction tools, including SIFT, PolyPhen-2, and MutationTaster. Given the location of the variant at the canonical splice donor site, its impact on mRNA processing was specifically evaluated using SpliceAI (https://spliceailookup.broadinstitute.org/) and another online prediction tool (https://rddc.tsinghua-gd.org/zh/tool/).

### 2.3 Functional validation experiment

Peripheral blood lymphocytes were isolated from anticoagulated whole blood samples (2 mL) using a lymphocyte separation medium (P8610; Solarbio; Beijing; China). Total RNA was extracted from the resulting lymphocytes using TRIzol reagent (DP424; Tiangen Biotech; Beijing; China). The reverse transcription reaction was carried out to synthesize the first-strand cDNA using the FastKing gDNA Dispelling RT SuperMix kit (KR118; Tiangen Biotech; Beijing; China). The cDNA was thereafter amplified by PCR using a commercial kit (KT211; Tiangen Biotech; Beijing; China). The gene-specific primers (forward: tgaaatgcttggcgactcct, and reverse: caggctctcctcctcctcat) were synthesized by Beijing Qingke Biotechnology Co., Ltd., Kunming Branch, China. The resulting DNA fragments were separated on a 1% agarose gel via electrophoresis. All experimental procedures described above were conducted strictly according to the manufacturer's instructions.

## 3 Case presentation

### 3.1 Clinical characteristics of the patient

The patient was a 6-year-and-10-month-old girl with normal growth and developmental milestones. Three years prior, she underwent chest CT for cough and sputum, which incidentally revealed a cystic lesion in the left upper lobe. Regular follow-up was performed without specific intervention. Recent CT showed an increase in the size of the cystic lesion, and the patient was admitted for further evaluation.

**Figure 1:**
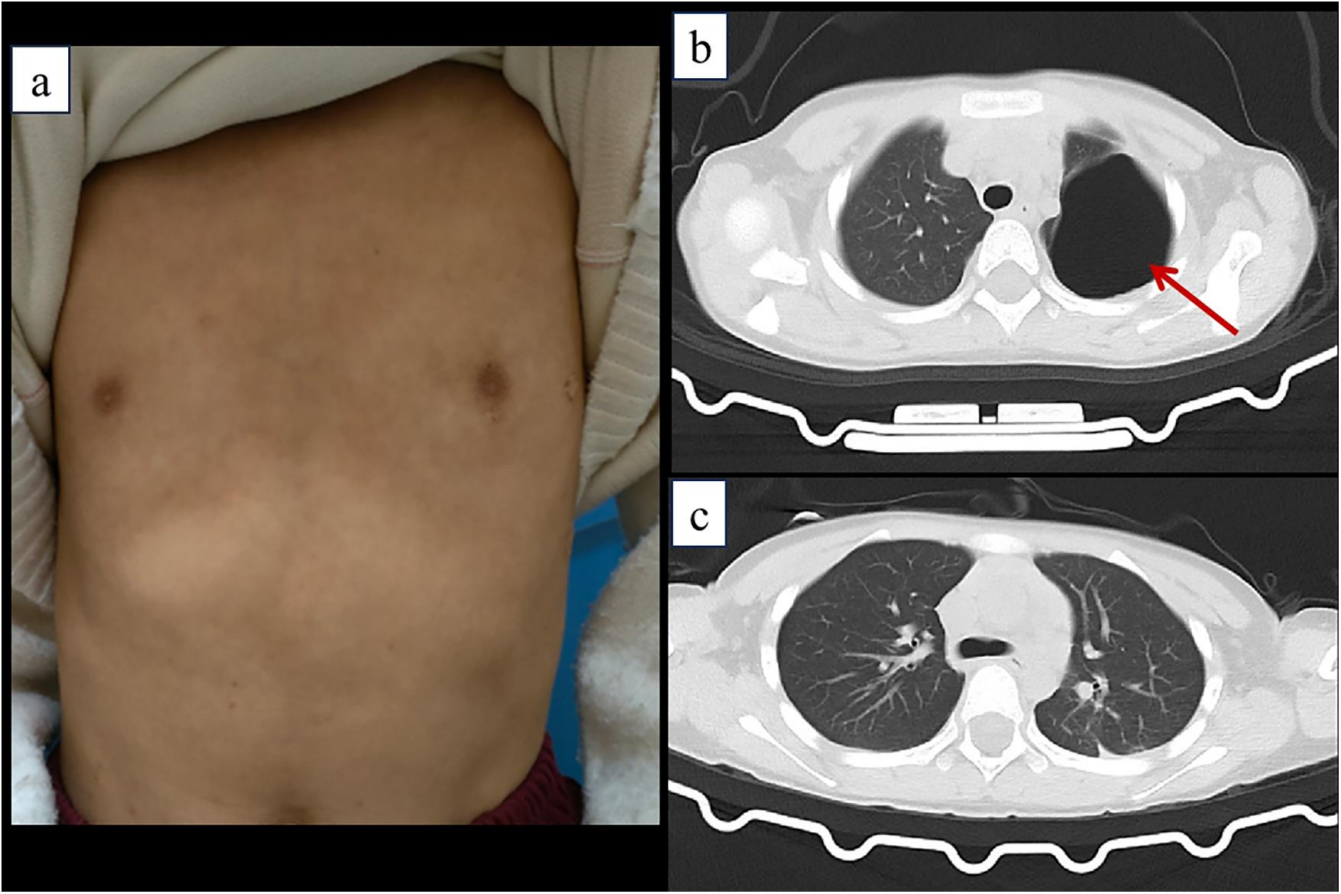
Chest examination of the patient. **(a)** Visual examination of the patient’ chest showed that the child's chest is flat with a depression in the lower part of the sternum and bilateral rib margins outward, consistent with the sign of funnel chest. **(b)** Enhanced CT scan showed a circular translucent lesion (red arrow) in the upper lobe of the left lung, with a thin wall and no lung markings inside. There were signs of adjacent lung tissue compression. **(c)** Follow-up chest CT after surgery showed a small cystic lucency with surrounding punctate calcifications at the left upper lobe surgical site and linear fibrotic changes in the left lower lobe.

Her medical history was notable for tonsillectomy due to adenoid hypertrophy 2 years earlier, with no other significant findings. At admission, she had no fever, cough, dyspnea, or other respiratory symptoms, and her diet and bowel movements were normal. Physical examination revealed stable vital signs. The chest was visually flat with a depressed lower sternum and outwardly flared bilateral costal margins, suggestive of pectus excavatum (Figure 1a). Auscultation demonstrated decreased breath sounds over the left upper lung and slightly increased breath sounds elsewhere.

**Figure 2:**
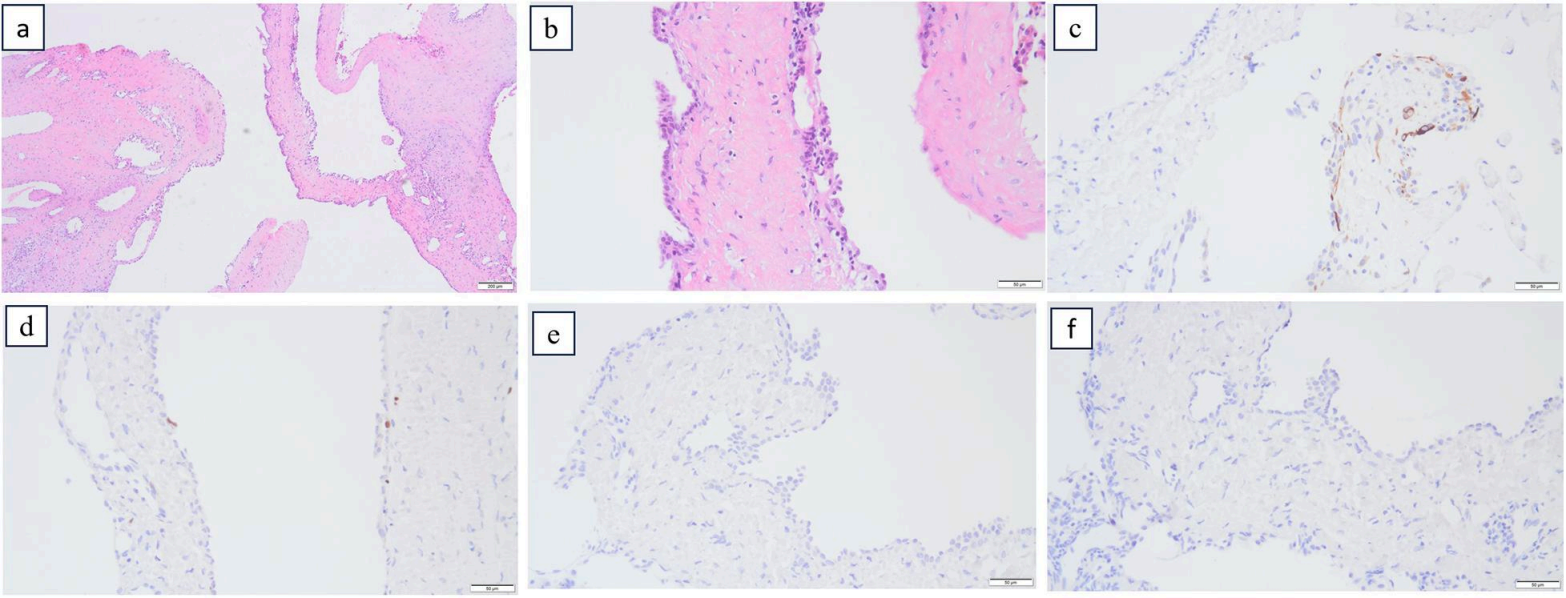
Postoperative histopathological examination. **(a)** HE staining (40×) reveals cystic spaces of variable dimensions within the lesion. **(b)** HE staining (200×) demonstrates ciliated columnar or cuboidal epithelium lining the capsule. **(c)** Desmin immunohistochemistry demonstrated sparse smooth muscle tissue in the cyst wall. **(d)** Immunohistochemistry for Ki67 demonstrated nuclear positivity in rare lining epithelial cells. **(e)** Immunohistochemistry for Myogenin showed negative results. **(f)** Immunohistochemistry for MyoD1 showed negative results.

Enhanced chest CT and 3D reconstruction revealed a large gas-filled cystic lesion in the left upper lobe (Figure 1b), and the preliminary diagnosis was CPAM IV with pectus excavatum. After completing preoperative evaluation, the patient underwent thoracoscopic wedge resection of the left upper lobe. Postoperative pathological histological analysis was performed in the Department of Pathology of our hospital. The surgically resected tissue measured 3.5 cm × 2 cm × 0.5 cm. It was divided into two parts, embedded in paraffin wax blocks, sectioned, and stained. All tissue sections were evaluated microscopically, which showed irregular thin-walled cysts lined by respiratory epithelium with widened interstitial stroma (Figures 2a,b). Immunohistochemical analysis revealed a small amount of Desmin-positive smooth muscle tissue within the cyst wall (Figure 2c), along with Ki67 positivity in a very few (approximately 1%) lining epithelial cells (Figure 2d). Staining for both Myogenin and MyoD1 was negative (Figures 2e,f). Collectively, these results are consistent with a diagnosis of CPAM IV.

### 3.2 Genetic analysis of whole-exome sequencing

**Figure 3:**
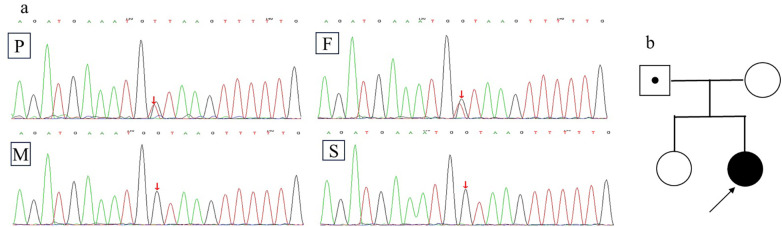
Genetic testing of the family. **(a)** Sanger sequencing of the variant (NM_ 177438.3 c.4206 + 1G > T). The patient (P) and her father (F) are heterozygous, and her mother (M) and sister (S) are normal. **(b)** The pedigree chart.

WES performed on the genomic DNA extracted from the surgically resected lung cyst tissue of the proband identified a heterozygous splicing variant in the *DICER1* gene (NM_177438.2: c.4206 + 1G > T, variant allele frequency 0.71). Sanger sequencing performed on peripheral blood demonstrated that the variant originated from the patient's father. however, the father remained clinically asymptomatic. Her mother and older sister do not carry the variant (Figure 3a). Pedigree analysis supported an autosomal dominant inheritance pattern with incomplete penetrance (Figure 3b). Additionally, a somatic *DICER1* hotspot mutation (c.5438A > G p.E1813G; variant allele frequency 0.16) was detected in the patient's tissue, consistent with the two-hit tumorigenesis model. No other tumor susceptibility-related variants were detected in this patient (Supplementary Table S1).

### 3.3 Bioinformatic analysis of variants

The identified c.4206 + 1G > T variant affects the highly conserved canonical donor splice site of intron 22 in the *DICER1* gene. In silico splicing analysis using the SpliceAI yielded a high delta splice loss score of 0.89, indicating a significant probability of normal splicing disruption. The online prediction tool showed that this variation would lead to a complete skipping of exon 22, which is 156 bp in length (Supplementary Figure S1).

### 3.4 Functional validation experiment

**Figure 4:**
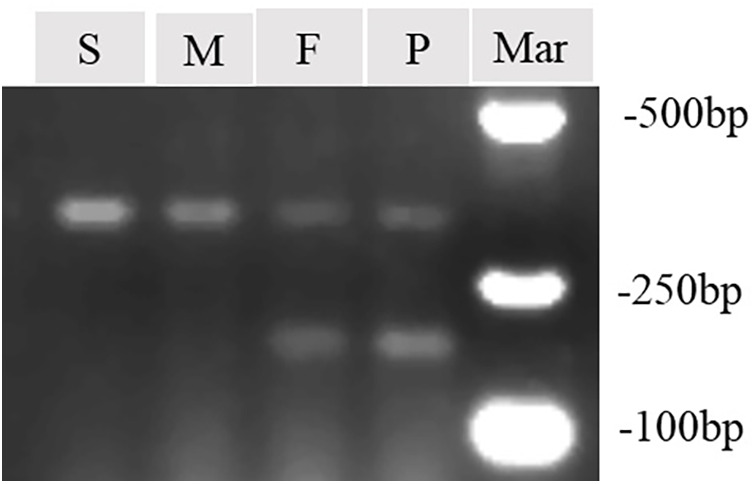
RT-PCR analysis of the variant. Agarose gel electrophoresis of the RT-PCR products revealed a significantly aberrant band (about 220 bp) in the patient (P) and her father (F), while in the unaffected mother (M) and sister (S) exhibited a single wild-type band (approximately 380 bp).

To verify the impact of the c.4206 + 1G > T variant on mRNA splicing, RT-PCR analysis was performed using RNA extracted from the peripheral blood of the family members. Agarose gel electrophoresis of the RT-PCR products revealed a single wild-type band (approximately 380 bp) in the unaffected mother (M) and sister (S). In contrast, both the proband (P) and her father (F) exhibited a characteristic dual-band pattern, consisting of the normal 380 bp band and an additional aberrant band of approximately 220 bp (Figure 4). The subsequent Sanger sequencing experiment failed because the PCR products of the patient and her father had degraded after being stored at −20°C for 2 months.

### 3.5 Follow-up

At 22 months of postoperative follow-up, the patient remained asymptomatic with no respiratory complaints. Follow-up chest CT demonstrated a small cystic lucency with surrounding punctate calcifications in the surgical area of the left upper lobe (Figure 1c), along with linear fibrotic changes in the left lower lobe. The clinical significance of this cystic finding remains uncertain and may be attributable to postoperative structural remodeling, low-grade DICER1-associated cystic change, or subclinical PPB-like evolution. At present, the patient remains clinically stable, with no evidence of tumor recurrence or extrapulmonary involvement, and is undergoing close radiological surveillance.

## 4 Discussion

We report a child initially diagnosed with CPAM IV who was found to carry a pathogenic germline splicing mutation in the *DICER1* gene (c.4206 + 1G > T). The *DICER1* gene, located on chromosome 14q32.13, encodes an RNase III endoribonuclease essential for microRNA (miRNA) biogenesis, thereby regulating post-transcriptional gene expression and fundamental cellular processes, including proliferation, differentiation, and apoptosis (14). Pathogenic variations in the *DICER1* predispose individuals to DICER1 tumor predisposition syndrome, an autosomal dominant condition in which PPB represents the hallmark tumor type (5).

According to the ACMG/AMP guidelines, the *DICER1* c.4206 + 1G > T variant identified in this study was classified as “Likely pathogenic”. The variant was not recorded in gnomAD (https://gnomad.broadinstitute.org/, v4.1.1), meets the PM2_supporting evidence criterion. Loss-of-function (LOF) is the pathogenic mechanism of DICER1 syndrome (15). This variant located at the +1 position of a canonical splice site. In silico splicing prediction indicated complete skipping of exon 22 (156 bp), which was subsequently confirmed by RT-PCR analysis of peripheral blood RNA from the patient and family members. The in-frame indels p.(Val1351_Met1402del) was outside of the RNase IIIb domain and repeat regions (https://cspec.genome.network/cspec/ui/svi/doc/GN024?version=1.4.0), supporting evidence level PVS1_moderate and PP3. This variant has been documented in a 35-year-old female with hereditary tumor predisposition syndrome, PS4_supporting evidence. Functional analysis demonstrated that the variant abrogates the function of *DICER1*, although another germline variant (CHEK2 c.1427C > T) was detected (16). Despite being an in-frame deletion, loss of exon 22 removes a sequence adjacent to the RNase IIIa catalytic domain, potentially impacting enzyme function, PS3_moderate evidence. Absence of these residues resulted in a very unstable protein expression (16). This structural defect blocks the normal processing of precursor miRNAs, leading to an imbalanced expression profile of mature miRNAs and dysregulation of downstream oncogenes (17).

The DICER1-related tumor susceptibility syndrome exemplifies an alternative two-hit model: one germline LOF variant, plus a second somatic missense variant in the RNase IIIb domain of the wild-type allele (in trans) (15). Consistent with the two-hit tumorigenesis model, we detected both a germline *DICER1* mutation (c.4206 + 1G > T) and a somatic hotspot *DICER1* mutation (c.5438A > G p.E1813G) in this case. Brcic et al. reported a lesion initially classified as CPAM type IV that harbored a somatic *DICER1* missense mutation (p.E1813K). The mutation was detected using a Cancer Hotspot Panel (covering 50 cancer-associated genes) and a custom panel covering all *DICER1* exons. Notably, this lesion exhibited rare MyoD-positive rhabdomyoblastic cells within the cystic stroma, a characteristic feature of early-stage PPB (18). These observations support the hypothesis that CPAM IV lesions may acquire somatic *DICER1* alterations and have the potential to progress along the PPB disease spectrum. However, neither MyoD1-positive nor Myogenin-positive cells were observed in our study. The only positive findings were a small number of Desmin-positive smooth muscle cells and occasional Ki67-positive cells. All findings support the diagnosis of CPAM IV. The RNase IIIb domain is critical for strand selection during miRNA processing. Hotspot mutations in this region can lead to defective miRNA processing and reverse-strand expression bias (19). In contrast, the germline splicing variant (c.4206 + 1G > T) identified in the present case results in persistent exon 22 skipping, affecting the RNase IIIa catalytic domain (16). Although differing in timing and molecular consequences, both variants are predicted to impair *DICER1* function (20). Consistent with the two-hit model, a germline mutation confers susceptibility, whereas the eventual development of a tumor typically requires acquisition of a second somatic mutation within the same cellular lineage (19). The diagnostic difficulty in this case reflects the core controversy in the academic community regarding the relationship between CPAM IV and PPB I.

A *DICER1* mutation carrier from a Chinese PPB family, initially diagnosed with CPAM type IV without malignant findings, later developed thyroid nodules 6 years post-surgery. This case illustrates the multi-organ tumor susceptibility associated with *DICER1* mutations (21). Therefore, regardless of whether the final pathological diagnosis is CPAM type IV or PPB type I, the coexistence of a pulmonary cystic lesion with both a germline mutation and a somatic hotspot mutation in *DICER1* may shift the clinical nature toward a tumor-predisposed state. Based on clinical recommendations and guidelines from international authoritative institutions, such as the PPB/DICER1 Registry (22, 23), individuals carrying pathogenic or likely pathogenic *DICER1* variants who develop pulmonary cystic lesions should be highly suspected of having PPB-spectrum disease until proven otherwise. Clinical management is recommended to adopt a comprehensive strategy including surgical evaluation, complete pathological examination, and initiation of multi-organ systemic tumor surveillance (22).

Sanger sequencing verification revealed that the variant was inherited from an asymptomatic father, consistent with the hallmark features of DICER1 syndrome, namely incomplete penetrance. Although the father is currently unaffected, each offspring carries a 50% genetic risk, underscoring the importance of genetic testing and counseling within the affected families. Based on current expert consensus and recommendations from the PPB/DICER1 Registry (24), we suggest an age-stratified surveillance strategy for such families. For pediatric patients, even after complete surgical resection of pulmonary lesions, continued chest imaging surveillance is still recommended.

### 4.1 Limitations

This study has several limitations. First, Sanger sequencing failed as the PCR products from the patient and her father had degraded. Therefore, the precise impact of this variant on *DICER1* pre-mRNA splicing could not be verified. Second, due to the limited follow-up period, whether the patient will subsequently develop tumor predisposition syndrome cannot be determined at this time. Future research should explore the impact of this variant on DICER1 protein stability and subsequent microRNA biosynthesis. Additionally, ongoing follow-up of this child will include tumor surveillance and genetic counseling.

## 5 Conclusion

In summary, this representative case illustrates the pivotal role of *DICER1* mutations in a subset of congenital pulmonary cystic lesions, particularly those resembling CPAM type IV. We support the view that DICER1-associated CPAM IV lesions could be regarded as an early and indolent manifestation within the PPB disease spectrum. Accordingly, for children diagnosed with CPAM IV based on imaging or pathological findings, regardless of family history, *DICER1* genetic testing could be considered, as it is essential for improving diagnostic accuracy, informing surgical management, and facilitating risk-adapted long-term surveillance.

## Data Availability

The raw sequence data reported in this paper have been deposited in the Genome Sequence Archive (Genomics, Proteomics & Bioinformatics 2025) in National Genomics Data Center (Nucleic Acids Res 2025), China National Center for Bioinformation / Beijing Institute of Genomics, Chinese Academy of Sciences (GSA-Human: HRA019494) that are publicly accessible at https://ngdc.cncb.ac.cn/gsa-human (25, 26).
