# Supplementary material for: Case Report: Congenital pulmonary airway malformation associated with a germline DICER1 splicing variant
Source: Front Pediatr. 2026 Jul 17;14:1876103. doi: 10.3389/fped.2026.1876103 (PMC13424480; doi:10.3389/fped.2026.1876103)
Supplement: Supplementary file 1 [file Datasheet1.pdf]

Supplementary Material

1 Supplementary Figure

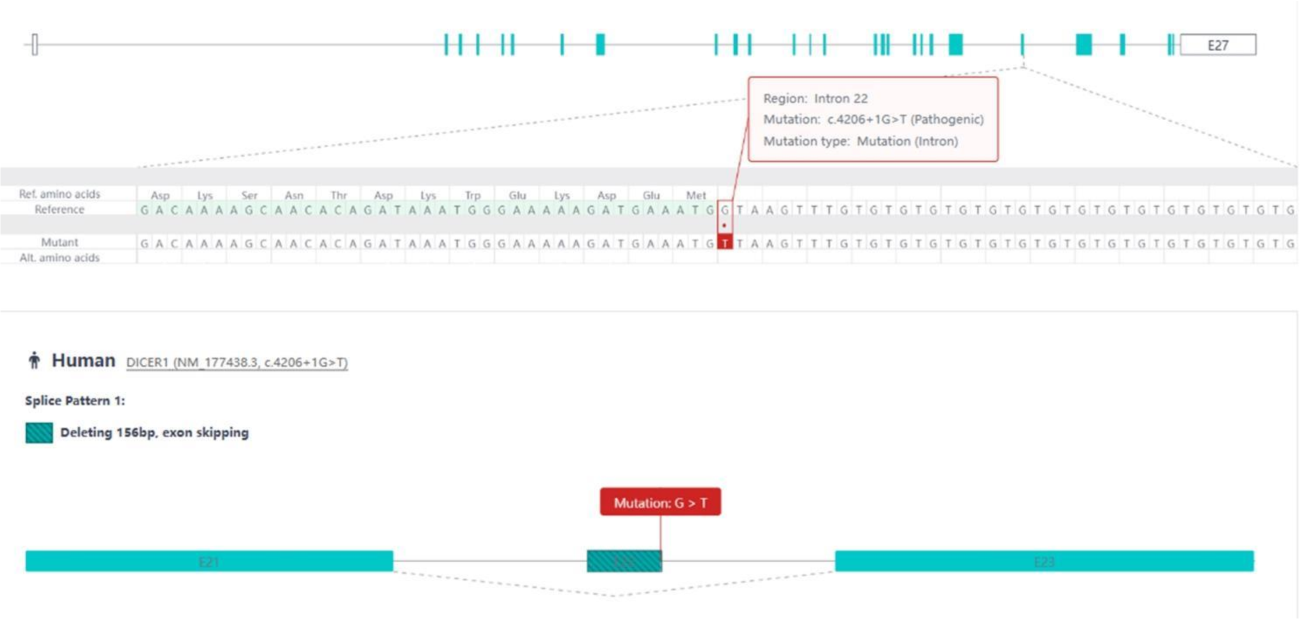

Supplementary Figure 1. The online prediction tool showed that the variant lead to a complete skipping of exon 22, which is 156 bp in length**.**

2 Supplementary Table

Table S1 Other variations detected in the proband.

Gene	Transcript exon	Nucleotide Amino Acids	Homozygous/ Heterozygous	Allele frequency in healthy individuals	Prediction	Pathogenicity Analysis (Score)	Disease/Phenotype (Inheritance Pattern)
HPS1	NM_000195.5;exon11	c.972dup(p.Met325HisfsTer128)	het	0.011	-	Pathogenic	Hermansky-Pudlak syndrome type 1 (AR)
SLC7A7	NM_003982.4;intron4	c.770+1G>A(p.?)	het	-	-	Likely pathogenic	Lysinuria-associated protein intolerance (AR)
LMF1	NM_022773.4;exon2	c.291C>A(p.Tyr97Ter)	het	-	-	Likely pathogenic	Combined lipase deficiency (AR)
ASL	NM_000048.4;exon17	c.1340G>C(p.Ser447Thr)	het	-	LD	Likely pathogenic	Argininosuccinic aciduria (AR)
UGT1A1	NM_000463.3;exon1	c.211G>A(p.Gly71Arg)	het	0.1538	LB	Likely pathogenic	1. [Gilbert syndrome] (AR) 2. Familial transient neonatal hyperbilirubinemia (AD, AR) 3. Crigler-Najjar syndrome type 2 (AR) 4. Crigler-Najjar syndrome type 1 (AR) 5. [Serum bilirubin level QTL1] (-)
PKP2	NM_001005242.3;exon3	c.369G>T(p.Trp123Cys)	het	-	U	Uncertain(1)	Arrhythmogenic Right Ventricular Dysplasia/Myocardopathy Type 9 (AD)
ALK	NM_004304.5;intron6	c.1415-1G>A(p.?)	het	0.0000082	-	Uncertain(1)	{Susceptible neuroblastoma 3} (-)
NSD1	NM_022455.5;exon5	c.3284G>A(p.Gly1095Asp)	het	-	LB	Uncertain(1)	Sotos syndrome (AD)
FGF23	NM_020638.3;exon3	c.340A>G(p.Arg114Gly)	het	0.000109	U	Uncertain(1)	1. Familial tumor-related calcosis, hyperphosphatemia type 2 (AR) 2. Autosomal dominant hypophosphatemic rickets (AD)
TIMP3	NM_000362.5;exon4	c.322G>A(p.Val108Ile)	het	0.0000082	U	Uncertain(1)	Sorsby retinal dystrophy (AD)
NFKB2	NM_001322934.2;exon14	c.1378G>T(p.Ala460Ser)	het	-	LB	Uncertain(1)	Common variant immunodeficiency type 10 (AD)
FBN2	NM_001999.4;exon11	c.1510C>A(p.Leu504Ile)	het	-	U	Uncertain(1)	1. Congenital contracture spider finger syndrome (AD) 2. Early-onset macular disease (AD)
CDH2	NM_001792.5;exon5	c.640C>G(p.Pro214Ala)	het	0.0001	U	Uncertain(1)	1. Attention deficit hyperactivity disorder type 8 (AD) 2. Arrhythmogenic right ventricular dysplasia type 14 (AD) 3. Corpus callosum dysgenesis-cardiac-eye-genital syndrome (AD)
APC	NM_000038.6;exon16	c.7049C>A(p.Ser2350Tyr)	het	-	U	Uncertain(1)	1. Hereditary fibrous dysplasia (AD) 2. Gardner syndrome (AD) 3. Gastric adenocarcinoma and proximal gastric polyposis (AD) 4. Periventricular adenoma somatic type (-) 5. Brain tumor polypoid syndrome type 2 (AD) 6. Familial adenomatous polyposis type 1 (AD)
RYR3	NM_001036.6;exon99	c.14129A>G(p.Asp4710Gly)	het	-	D	Uncertain(5)	Congenital myopathy type 20 (AR)
RYR3	NM_001036.6;exon35	c.4961G>A(p.Gly1654Glu)	het	0.000641	LD	Uncertain(2)	Congenital myopathy type 20 (AR)
CAPN3	NM_000070.3;exon13	c.1575C>G(p.Phe525Leu)	het	0.000641	U	Uncertain(3)	1. Limb-girdle muscular dystrophy type 1, autosomal recessive inheritance (AR) 2.

							Limb-girdle muscular dystrophy type 4, autosomal dominant inheritance (AD)
CCDC88C	NM_001080414.4;exon15	c.2630_2668del(p.Ala877_Leu889del)	het	0.0000083	-	Uncertain(3)	1. Spinal-cerebellar ataxia type 40 (AD) 2. Congenital hydrocephalus type 1 (AR)
STUB1	NM_005861.4;exon7	c.889A>T(p.Asn297Tyr)	het	-	U	Uncertain(1)	1. Spinal cerebellar ataxia type 48 (AD) 2. Autosomal recessive spinal cerebellar ataxia type 16 (AR)
ANXA11	NM_145868.2;exon9	c.887T>A (p.Ile296Asn)	het	0.0001	U	Uncertain(0)	1. Inclusion body myopathy and leukoencephalopathy (AD) 2. Muscular atrophy lateral sclerosis type 23 (AD)
PKD1	NM_001009944.3;exon6	c.1305G>C(p.Gln435His)	het	-	LB	Uncertain(-1)	Polycystic kidney disease type 1 (AD)
COL4A3	NM_000091.5;exon28	c.2087T>C(p.Ile696Thr)	het	-	U	Uncertain(1)	1. Autosomal recessive Alport syndrome type 3B (AR) 2. Benign familial hematuria type 2 (AD) 3. Autosomal dominant Alport syndrome type 3A (ATS3A) (AD)
FLNB	NM_001457.4;exon36	c.5917G>A(p.Glu1973Lys)	het	0.0000478	LD	Uncertain(2)	1. Boomerang dysplasia (AD) 2. Osteogenesis imperfecta type 3 (AD) 3. Osteogenesis imperfecta type 1 (AD) 4. Larsen syndrome (AD) 5. Vertebral sac-bone junction syndrome (AR)
MEFV	NM_000243.3;exon3	c.1223G>A(p.Arg408Gln)	het	0.0634615	LB	Uncertain(0)	1. Autosomal dominant familial Mediterranean fever (AD) 2. Acute febrile neutrophilic dermatosis (AD) 3. Familial Mediterranean fever (AR)
MEFV	NM_000243.3;exon3	c.1105C>T(p.Pro369Ser)	het	0.0886889	LB	Uncertain(0)	1. Autosomal dominant familial Mediterranean fever (AD) 2. Acute febrile neutrophilic dermatosis (AD) 3. Familial Mediterranean fever (AR)
GCLM	NM_002061.4;intron5	c.541-15T>C(p.?)	het	-	-	Uncertain(0)	Myocardial infarction susceptibility type (-)
CHD6	NM_032221.5;exon31	c.4790A>G(p.Asp1597Gly)	het	-	D	Uncertain(4)	HALLERMANN-STREIFF syndrome (AD)
F8	NM_000132.4;exon14	c.3169G>A(p.Glu1057Lys)	het	0.008	LD	Uncertain(1)	1. Hemophilia A (X-linked recessive) 2. X-linked thrombosis 13 (XLD) caused by factor VIII deficiency

Note: Prediction, protein function was predicted using the REVEL software.

Abbreviations: het, heterozygous; LB, likely benign; D, damaging; LD, likely damaging; U, uncertain; AR, autosomal recessive; AD, autosomal dominant.
