# Supplementary figures and images for: Case Report: Congenital pulmonary airway malformation associated with a germline DICER1 splicing variant
Source: Front Pediatr. 2026 Jul 17;14:1876103. doi: 10.3389/fped.2026.1876103 (PMC13424480; doi:10.3389/fped.2026.1876103)

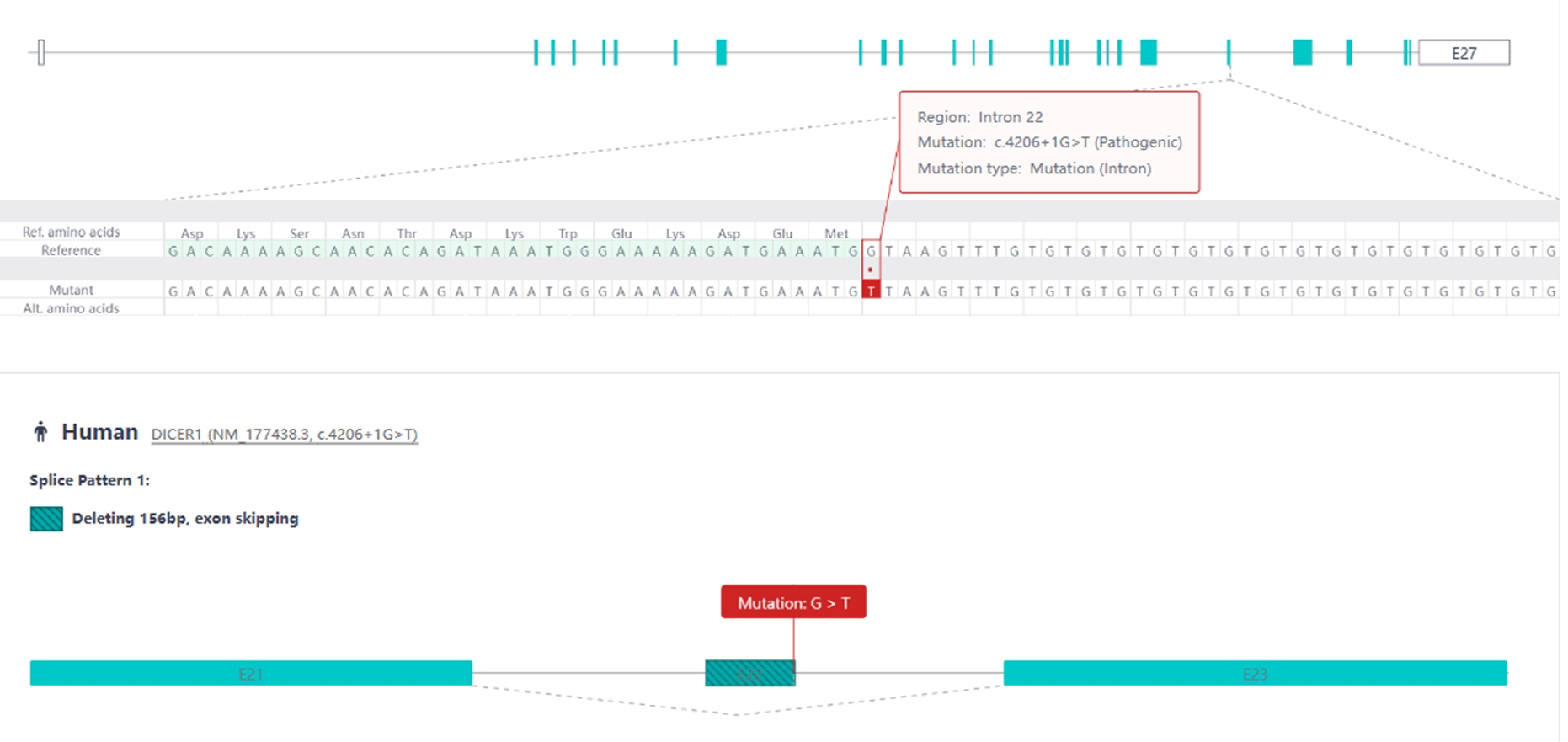

Supplement: Supplementary file 2 [file Image1.jpeg]
